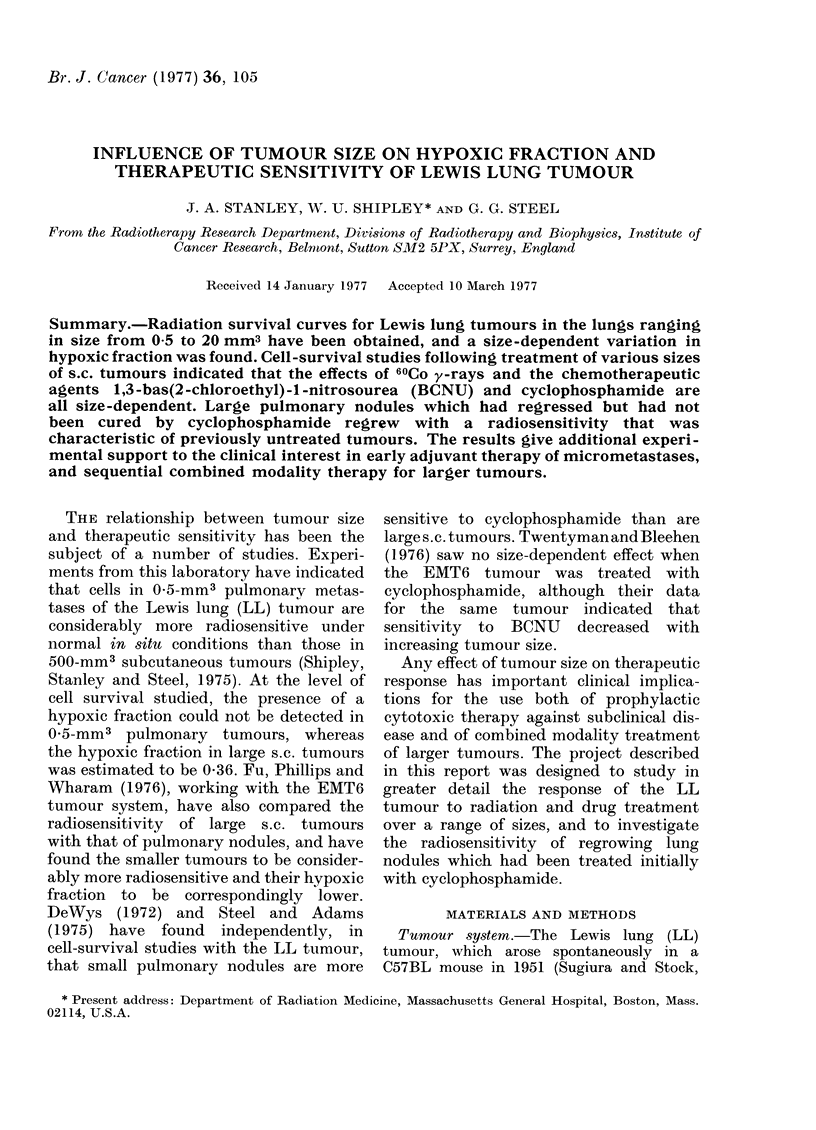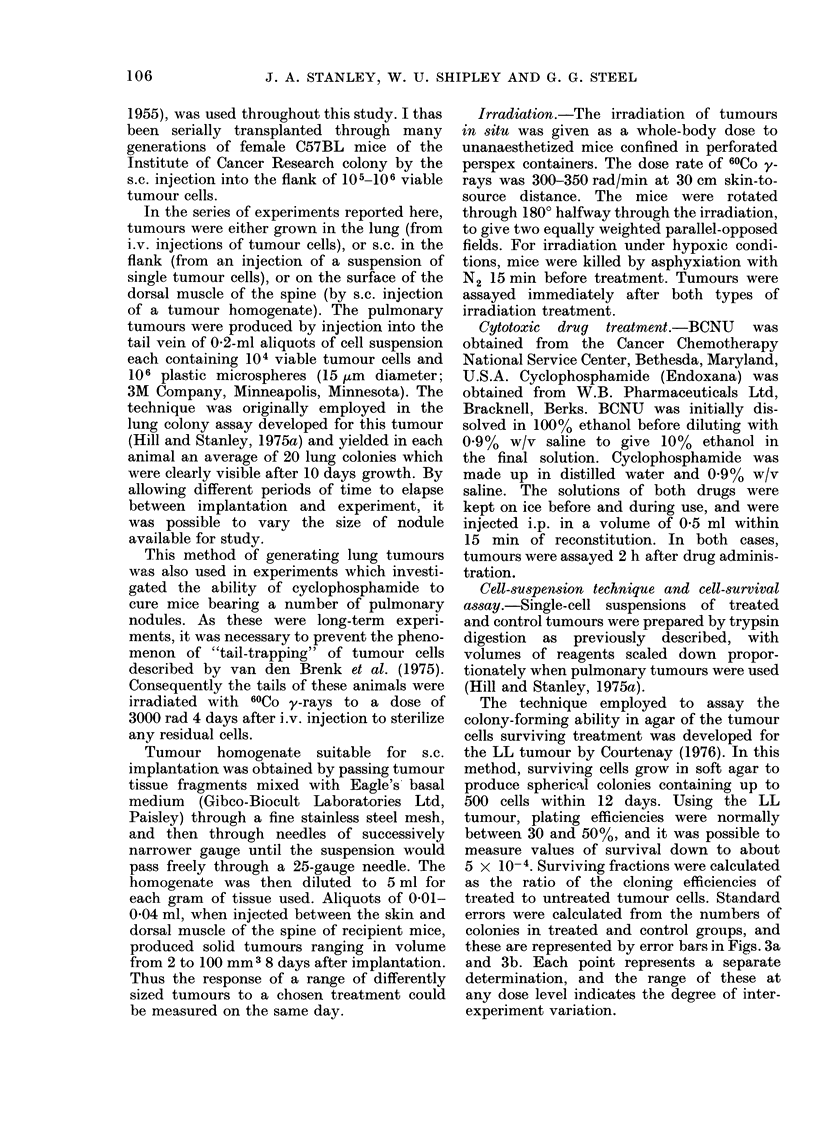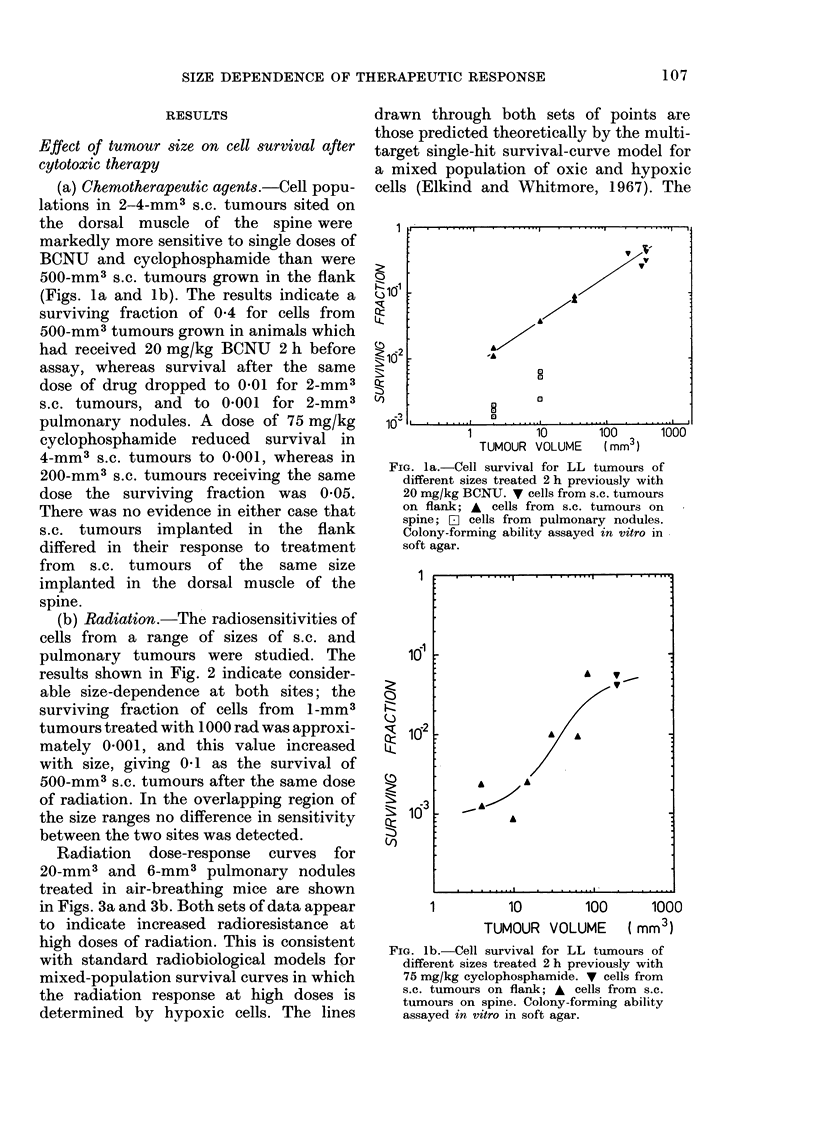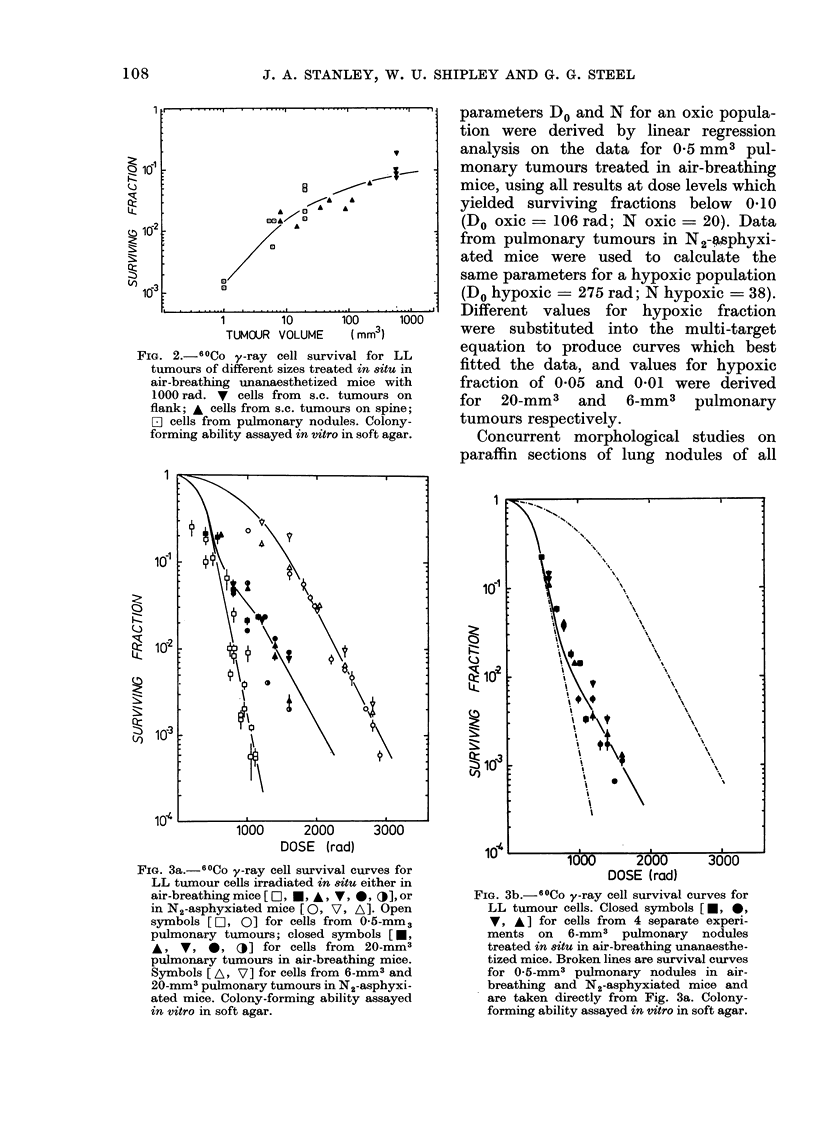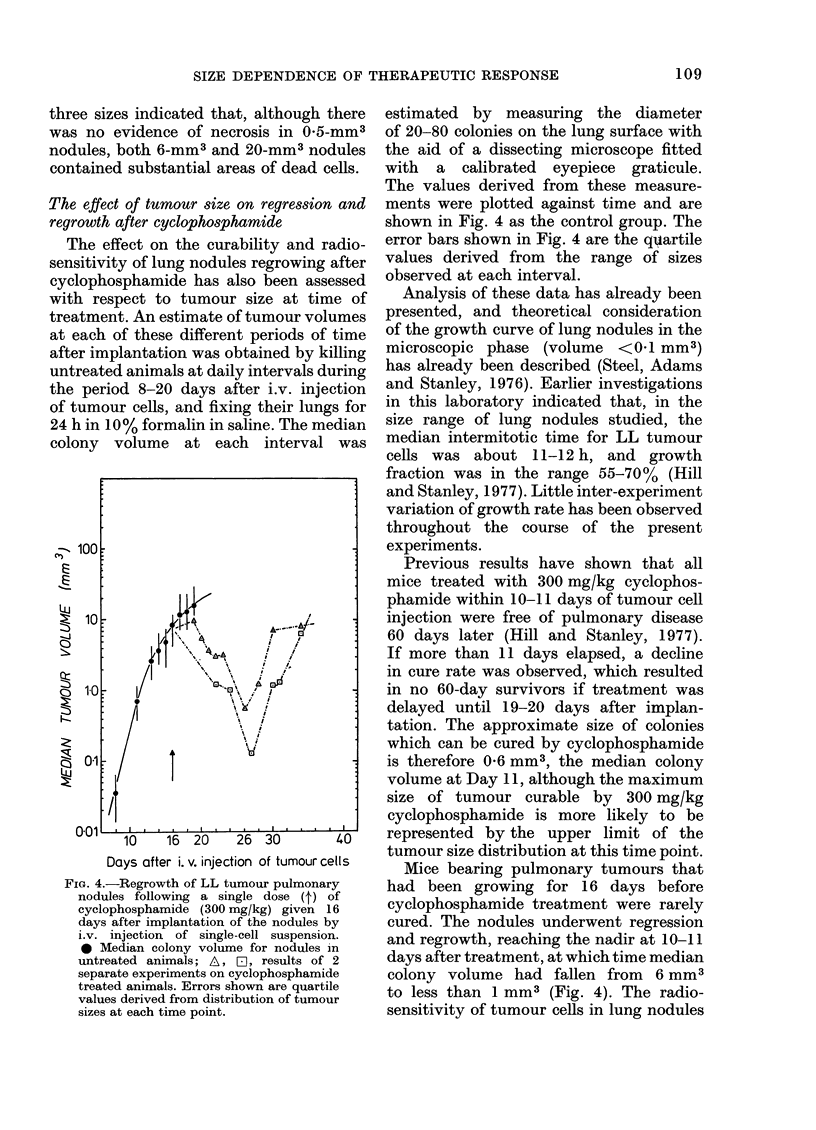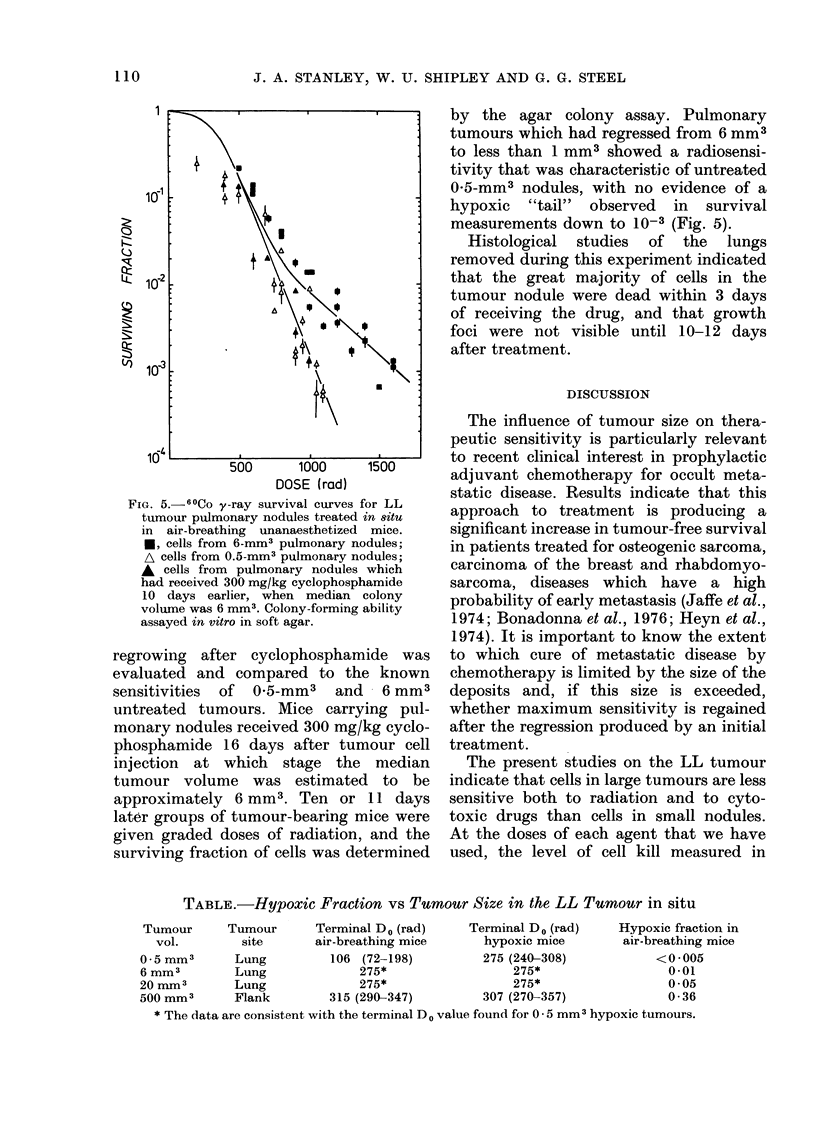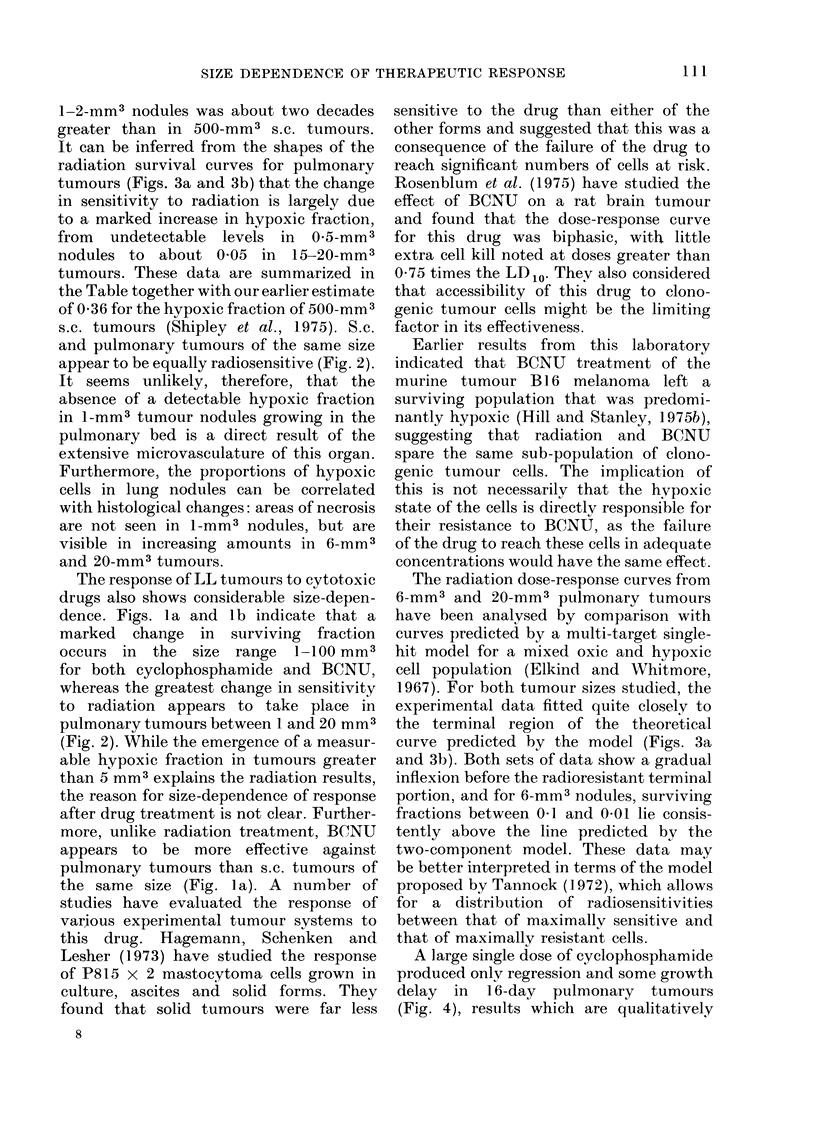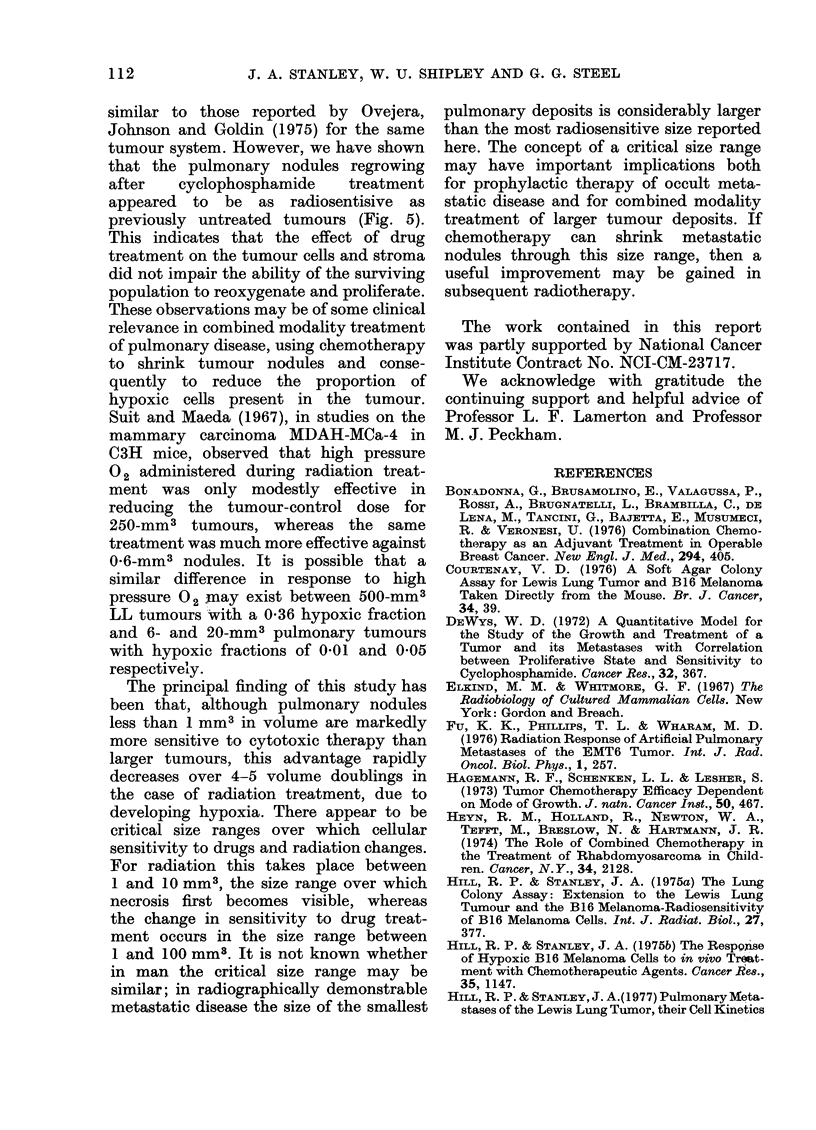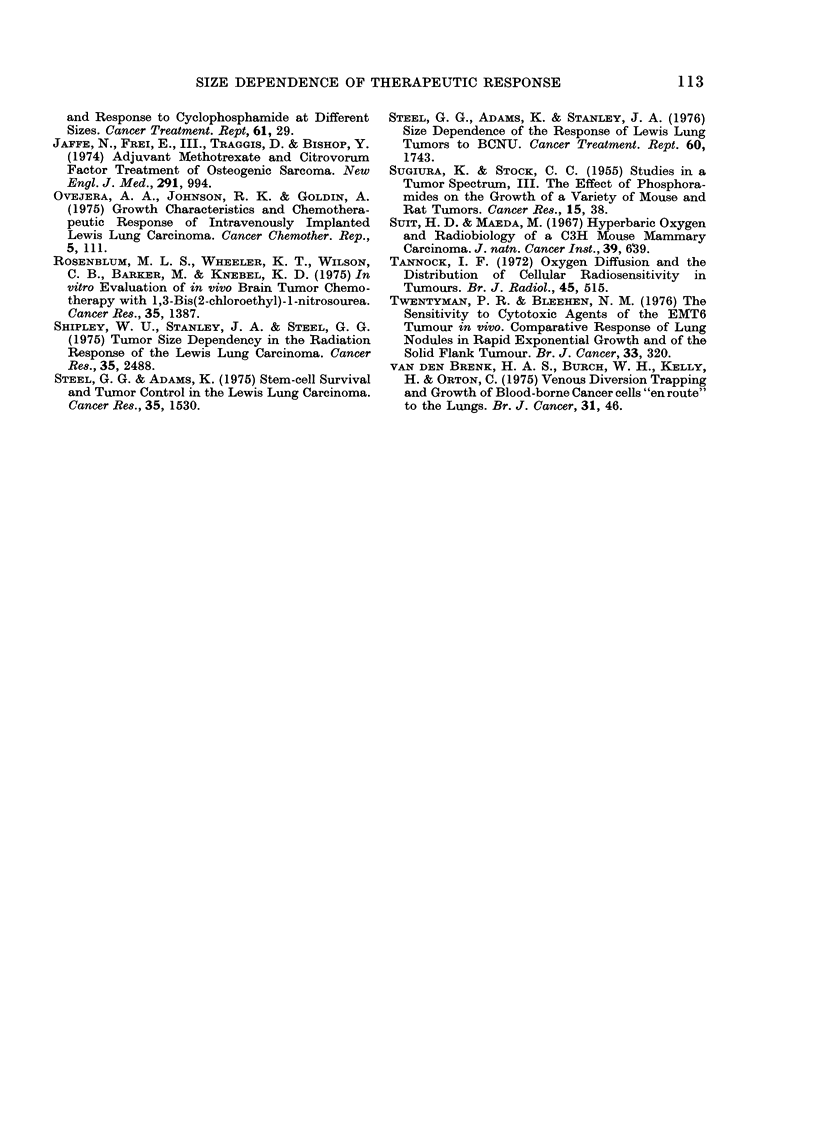# Influence of tumour size on hypoxic fraction and therapeutic sensitivity of Lewis lung tumour.

**DOI:** 10.1038/bjc.1977.160

**Published:** 1977-07

**Authors:** J. A. Stanley, W. U. Shipley, G. G. Steel

## Abstract

Radiation survival curves for Lewis lung tumours in the lungs ranging in size from 0-5 to 20 mm3 have been obtained, and a size-dependent variation in hypoxic fraction was found. Cell-survival studies following treatment of various sizes of s.c. tumours indicated that the effects of 60Co gamma-rays and the chemotherapeutic agents 1,3-bas(2-chloroethyl)-1-nitrosourea (BCNU) and cyclophosphamide are all size-dependent. Large pulmonary nodules which had regressed but had not been cured by cyclophosphamide regrew with a radiosensitivity that was characteristic of previously untreated tumours. The results give additional experimental support to the clinical interest in early adjuvant therapy of micrometastases, and sequential combined modality therapy for larger tumours.


*Br. J. Cancer* (1977) **36**, 105

### INFLUENCE OF TUMOUR SIZE ON HYPOXIC FRACTION AND THERAPEUTIC SENSITIVITY OF LEWIS LUNG TUMOUR

J. A. STANLEY, W. U. SHIPLEY* AND G. G. STEEL

*From the Radiotherapy Research Department, Divisions of Radiotherapy and Biophysics, Institute of Cancer Research, Belmont, Sutton SM2 5PX, Surrey, England*

Received 14 January 1977 Accepted 10 March 1977

**Summary.—Radiation survival curves for Lewis lung tumours in the lungs ranging in size from 0·5 to 20 mm$^3$ have been obtained, and a size-dependent variation in hypoxic fraction was found. Cell-survival studies following treatment of various sizes of s.c. tumours indicated that the effects of $^{60}$Co $\gamma$-rays and the chemotherapeutic agents 1,3-bas(2-chloroethyl)-1-nitrosourea (BCNU) and cyclophosphamide are all size-dependent. Large pulmonary nodules which had regressed but had not been cured by cyclophosphamide regrew with a radiosensitivity that was characteristic of previously untreated tumours. The results give additional experimental support to the clinical interest in early adjuvant therapy of micrometastases, and sequential combined modality therapy for larger tumours.**

THE relationship between tumour size and therapeutic sensitivity has been the subject of a number of studies. Experiments from this laboratory have indicated that cells in 0·5-mm$^3$ pulmonary metastases of the Lewis lung (LL) tumour are considerably more radiosensitive under normal *in situ* conditions than those in 500-mm$^3$ subcutaneous tumours (Shipley, Stanley and Steel, 1975). At the level of cell survival studied, the presence of a hypoxic fraction could not be detected in 0·5-mm$^3$ pulmonary tumours, whereas the hypoxic fraction in large s.c. tumours was estimated to be 0·36. Fu, Phillips and Wharam (1976), working with the EMT6 tumour system, have also compared the radiosensitivity of large s.c. tumours with that of pulmonary nodules, and have found the smaller tumours to be considerably more radiosensitive and their hypoxic fraction to be correspondingly lower. DeWys (1972) and Steel and Adams (1975) have found independently, in cell-survival studies with the LL tumour, that small pulmonary nodules are more sensitive to cyclophosphamide than are large s.c. tumours. Twentyman and Bleehen (1976) saw no size-dependent effect when the EMT6 tumour was treated with cyclophosphamide, although their data for the same tumour indicated that sensitivity to BCNU decreased with increasing tumour size.

Any effect of tumour size on therapeutic response has important clinical implications for the use both of prophylactic cytotoxic therapy against subclinical disease and of combined modality treatment of larger tumours. The project described in this report was designed to study in greater detail the response of the LL tumour to radiation and drug treatment over a range of sizes, and to investigate the radiosensitivity of regrowing lung nodules which had been treated initially with cyclophosphamide.

### MATERIALS AND METHODS

*Tumour system.*—The Lewis lung (LL) tumour, which arose spontaneously in a C57BL mouse in 1951 (Sugiura and Stock,

* Present address: Department of Radiation Medicine, Massachusetts General Hospital, Boston, Mass. 02114, U.S.A.

1955), was used throughout this study. I thas been serially transplanted through many generations of female C57BL mice of the Institute of Cancer Research colony by the s.c. injection into the flank of $10^5$–$10^6$ viable tumour cells.

In the series of experiments reported here, tumours were either grown in the lung (from i.v. injections of tumour cells), or s.c. in the flank (from an injection of a suspension of single tumour cells), or on the surface of the dorsal muscle of the spine (by s.c. injection of a tumour homogenate). The pulmonary tumours were produced by injection into the tail vein of 0·2-ml aliquots of cell suspension each containing $10^4$ viable tumour cells and $10^6$ plastic microspheres (15 $\mu$m diameter; 3M Company, Minneapolis, Minnesota). The technique was originally employed in the lung colony assay developed for this tumour (Hill and Stanley, 1975*a*) and yielded in each animal an average of 20 lung colonies which were clearly visible after 10 days growth. By allowing different periods of time to elapse between implantation and experiment, it was possible to vary the size of nodule available for study.

This method of generating lung tumours was also used in experiments which investigated the ability of cyclophosphamide to cure mice bearing a number of pulmonary nodules. As these were long-term experiments, it was necessary to prevent the phenomenon of "tail-trapping" of tumour cells described by van den Brenk *et al.* (1975). Consequently the tails of these animals were irradiated with $^{60}$Co $\gamma$-rays to a dose of 3000 rad 4 days after i.v. injection to sterilize any residual cells.

Tumour homogenate suitable for s.c. implantation was obtained by passing tumour tissue fragments mixed with Eagle's basal medium (Gibco-Biocult Laboratories Ltd, Paisley) through a fine stainless steel mesh, and then through needles of successively narrower gauge until the suspension would pass freely through a 25-gauge needle. The homogenate was then diluted to 5 ml for each gram of tissue used. Aliquots of 0·01–0·04 ml, when injected between the skin and dorsal muscle of the spine of recipient mice, produced solid tumours ranging in volume from 2 to 100 mm$^3$ 8 days after implantation. Thus the response of a range of differently sized tumours to a chosen treatment could be measured on the same day.

*Irradiation.*—The irradiation of tumours *in situ* was given as a whole-body dose to unanaesthetized mice confined in perforated perspex containers. The dose rate of $^{60}$Co $\gamma$-rays was 300–350 rad/min at 30 cm skin-to-source distance. The mice were rotated through 180° halfway through the irradiation, to give two equally weighted parallel-opposed fields. For irradiation under hypoxic conditions, mice were killed by asphyxiation with $N_2$ 15 min before treatment. Tumours were assayed immediately after both types of irradiation treatment.

*Cytotoxic drug treatment.*—BCNU was obtained from the Cancer Chemotherapy National Service Center, Bethesda, Maryland, U.S.A. Cyclophosphamide (Endoxana) was obtained from W.B. Pharmaceuticals Ltd, Bracknell, Berks. BCNU was initially dissolved in 100% ethanol before diluting with 0·9% w/v saline to give 10% ethanol in the final solution. Cyclophosphamide was made up in distilled water and 0·9% w/v saline. The solutions of both drugs were kept on ice before and during use, and were injected i.p. in a volume of 0·5 ml within 15 min of reconstitution. In both cases, tumours were assayed 2 h after drug administration.

*Cell-suspension technique and cell-survival assay.*—Single-cell suspensions of treated and control tumours were prepared by trypsin digestion as previously described, with volumes of reagents scaled down proportionately when pulmonary tumours were used (Hill and Stanley, 1975*a*).

The technique employed to assay the colony-forming ability in agar of the tumour cells surviving treatment was developed for the LL tumour by Courtenay (1976). In this method, surviving cells grow in soft agar to produce spherical colonies containing up to 500 cells within 12 days. Using the LL tumour, plating efficiencies were normally between 30 and 50%, and it was possible to measure values of survival down to about $5 \times 10^{-4}$. Surviving fractions were calculated as the ratio of the cloning efficiencies of treated to untreated tumour cells. Standard errors were calculated from the numbers of colonies in treated and control groups, and these are represented by error bars in Figs. 3a and 3b. Each point represents a separate determination, and the range of these at any dose level indicates the degree of inter-experiment variation.

### RESULTS

### *Effect of tumour size on cell survival after cytotoxic therapy*

(a) *Chemotherapeutic agents.*—Cell populations in 2–4-mm$^3$ s.c. tumours sited on the dorsal muscle of the spine were markedly more sensitive to single doses of BCNU and cyclophosphamide than were 500-mm$^3$ s.c. tumours grown in the flank (Figs. 1a and 1b). The results indicate a surviving fraction of 0·4 for cells from 500-mm$^3$ tumours grown in animals which had received 20 mg/kg BCNU 2 h before assay, whereas survival after the same dose of drug dropped to 0·01 for 2-mm$^3$ s.c. tumours, and to 0·001 for 2-mm$^3$ pulmonary nodules. A dose of 75 mg/kg cyclophosphamide reduced survival in 4-mm$^3$ s.c. tumours to 0·001, whereas in 200-mm$^3$ s.c. tumours receiving the same dose the surviving fraction was 0·05. There was no evidence in either case that s.c. tumours implanted in the flank differed in their response to treatment from s.c. tumours of the same size implanted in the dorsal muscle of the spine.

(b) *Radiation.*—The radiosensitivities of cells from a range of sizes of s.c. and pulmonary tumours were studied. The results shown in Fig. 2 indicate considerable size-dependence at both sites; the surviving fraction of cells from 1-mm$^3$ tumours treated with 1000 rad was approximately 0·001, and this value increased with size, giving 0·1 as the survival of 500-mm$^3$ s.c. tumours after the same dose of radiation. In the overlapping region of the size ranges no difference in sensitivity between the two sites was detected.

Radiation dose-response curves for 20-mm$^3$ and 6-mm$^3$ pulmonary nodules treated in air-breathing mice are shown in Figs. 3a and 3b. Both sets of data appear to indicate increased radioresistance at high doses of radiation. This is consistent with standard radiobiological models for mixed-population survival curves in which the radiation response at high doses is determined by hypoxic cells. The lines drawn through both sets of points are those predicted theoretically by the multi-target single-hit survival-curve model for a mixed population of oxic and hypoxic cells (Elkind and Whitmore, 1967). The

FIG. 1a.—Cell survival for LL tumours of different sizes treated 2 h previously with 20 mg/kg BCNU. ▼ cells from s.c. tumours on flank; ▲ cells from s.c. tumours on spine; ⊡ cells from pulmonary nodules. Colony-forming ability assayed *in vitro* in soft agar.

FIG. 1b.—Cell survival for LL tumours of different sizes treated 2 h previously with 75 mg/kg cyclophosphamide. ▼ cells from s.c. tumours on flank; ▲ cells from s.c. tumours on spine. Colony-forming ability assayed *in vitro* in soft agar.

FIG. 2.—$^{60}$Co $\gamma$-ray cell survival for LL tumours of different sizes treated *in situ* in air-breathing unanaesthetized mice with 1000 rad. ▼ cells from s.c. tumours on flank; ▲ cells from s.c. tumours on spine; □ cells from pulmonary nodules. Colony-forming ability assayed *in vitro* in soft agar.

parameters $D_0$ and N for an oxic population were derived by linear regression analysis on the data for 0·5 $mm^3$ pulmonary tumours treated in air-breathing mice, using all results at dose levels which yielded surviving fractions below 0·10 ($D_0$ oxic = 106 rad; N oxic = 20). Data from pulmonary tumours in $N_2$-asphyxiated mice were used to calculate the same parameters for a hypoxic population ($D_0$ hypoxic = 275 rad; N hypoxic = 38). Different values for hypoxic fraction were substituted into the multi-target equation to produce curves which best fitted the data, and values for hypoxic fraction of 0·05 and 0·01 were derived for 20-$mm^3$ and 6-$mm^3$ pulmonary tumours respectively.

Concurrent morphological studies on paraffin sections of lung nodules of all

FIG. 3a.—$^{60}$Co $\gamma$-ray cell survival curves for LL tumour cells irradiated *in situ* either in air-breathing mice [□, ■, ▲, ▼, ●, ◑], or in $N_2$-asphyxiated mice [○, ▽, △]. Open symbols [□, ○] for cells from 0·5-$mm_3$ pulmonary tumours; closed symbols [■, ▲, ▼, ●, ◑] for cells from 20-$mm^3$ pulmonary tumours in air-breathing mice. Symbols [△, ▽] for cells from 6-$mm^3$ and 20-$mm^3$ pulmonary tumours in $N_2$-asphyxiated mice. Colony-forming ability assayed *in vitro* in soft agar.

FIG. 3b.—$^{60}$Co $\gamma$-ray cell survival curves for LL tumour cells. Closed symbols [■, ●, ▼, ▲] for cells from 4 separate experiments on 6-$mm^3$ pulmonary nodules treated *in situ* in air-breathing unanaesthetized mice. Broken lines are survival curves for 0·5-$mm^3$ pulmonary nodules in air-breathing and $N_2$-asphyxiated mice and are taken directly from Fig. 3a. Colony-forming ability assayed *in vitro* in soft agar.

three sizes indicated that, although there was no evidence of necrosis in 0·5-mm$^3$ nodules, both 6-mm$^3$ and 20-mm$^3$ nodules contained substantial areas of dead cells.

*The effect of tumour size on regression and regrowth after cyclophosphamide*

The effect on the curability and radiosensitivity of lung nodules regrowing after cyclophosphamide has also been assessed with respect to tumour size at time of treatment. An estimate of tumour volumes at each of these different periods of time after implantation was obtained by killing untreated animals at daily intervals during the period 8–20 days after i.v. injection of tumour cells, and fixing their lungs for 24 h in 10% formalin in saline. The median colony volume at each interval was estimated by measuring the diameter of 20–80 colonies on the lung surface with the aid of a dissecting microscope fitted with a calibrated eyepiece graticule. The values derived from these measurements were plotted against time and are shown in Fig. 4 as the control group. The error bars shown in Fig. 4 are the quartile values derived from the range of sizes observed at each interval.

FIG. 4.—Regrowth of LL tumour pulmonary nodules following a single dose (↑) of cyclophosphamide (300 mg/kg) given 16 days after implantation of the nodules by i.v. injection of single-cell suspension. ● Median colony volume for nodules in untreated animals; △, ⊡, results of 2 separate experiments on cyclophosphamide treated animals. Errors shown are quartile values derived from distribution of tumour sizes at each time point.

Analysis of these data has already been presented, and theoretical consideration of the growth curve of lung nodules in the microscopic phase (volume $<0{\cdot}1$ mm$^3$) has already been described (Steel, Adams and Stanley, 1976). Earlier investigations in this laboratory indicated that, in the size range of lung nodules studied, the median intermitotic time for LL tumour cells was about 11–12 h, and growth fraction was in the range 55–70% (Hill and Stanley, 1977). Little inter-experiment variation of growth rate has been observed throughout the course of the present experiments.

Previous results have shown that all mice treated with 300 mg/kg cyclophosphamide within 10–11 days of tumour cell injection were free of pulmonary disease 60 days later (Hill and Stanley, 1977). If more than 11 days elapsed, a decline in cure rate was observed, which resulted in no 60-day survivors if treatment was delayed until 19–20 days after implantation. The approximate size of colonies which can be cured by cyclophosphamide is therefore 0·6 mm$^3$, the median colony volume at Day 11, although the maximum size of tumour curable by 300 mg/kg cyclophosphamide is more likely to be represented by the upper limit of the tumour size distribution at this time point.

Mice bearing pulmonary tumours that had been growing for 16 days before cyclophosphamide treatment were rarely cured. The nodules underwent regression and regrowth, reaching the nadir at 10–11 days after treatment, at which time median colony volume had fallen from 6 mm$^3$ to less than 1 mm$^3$ (Fig. 4). The radiosensitivity of tumour cells in lung nodules

FIG. 5.—$^{60}$Co $\gamma$-ray survival curves for LL tumour pulmonary nodules treated *in situ* in air-breathing unanaesthetized mice. ■, cells from 6-mm$^3$ pulmonary nodules; △ cells from 0.5-mm$^3$ pulmonary nodules; ▲ cells from pulmonary nodules which had received 300 mg/kg cyclophosphamide 10 days earlier, when median colony volume was 6 mm$^3$. Colony-forming ability assayed *in vitro* in soft agar.

regrowing after cyclophosphamide was evaluated and compared to the known sensitivities of 0·5-mm$^3$ and 6 mm$^3$ untreated tumours. Mice carrying pulmonary nodules received 300 mg/kg cyclophosphamide 16 days after tumour cell injection at which stage the median tumour volume was estimated to be approximately 6 mm$^3$. Ten or 11 days later groups of tumour-bearing mice were given graded doses of radiation, and the surviving fraction of cells was determined by the agar colony assay. Pulmonary tumours which had regressed from 6 mm$^3$ to less than 1 mm$^3$ showed a radiosensitivity that was characteristic of untreated 0·5-mm$^3$ nodules, with no evidence of a hypoxic "tail" observed in survival measurements down to $10^{-3}$ (Fig. 5).

Histological studies of the lungs removed during this experiment indicated that the great majority of cells in the tumour nodule were dead within 3 days of receiving the drug, and that growth foci were not visible until 10–12 days after treatment.

### DISCUSSION

The influence of tumour size on therapeutic sensitivity is particularly relevant to recent clinical interest in prophylactic adjuvant chemotherapy for occult metastatic disease. Results indicate that this approach to treatment is producing a significant increase in tumour-free survival in patients treated for osteogenic sarcoma, carcinoma of the breast and rhabdomyosarcoma, diseases which have a high probability of early metastasis (Jaffe *et al.*, 1974; Bonadonna *et al.*, 1976; Heyn *et al.*, 1974). It is important to know the extent to which cure of metastatic disease by chemotherapy is limited by the size of the deposits and, if this size is exceeded, whether maximum sensitivity is regained after the regression produced by an initial treatment.

The present studies on the LL tumour indicate that cells in large tumours are less sensitive both to radiation and to cytotoxic drugs than cells in small nodules. At the doses of each agent that we have used, the level of cell kill measured in

TABLE.—*Hypoxic Fraction* vs *Tumour Size in the LL Tumour* in situ

| Tumour vol. | Tumour site | Terminal $D_0$ (rad) air-breathing mice | Terminal $D_0$ (rad) hypoxic mice | Hypoxic fraction in air-breathing mice |
|---|---|---|---|---|
| 0·5 mm$^3$ | Lung | 106 (72–198) | 275 (240–308) | <0·005 |
| 6 mm$^3$ | Lung | 275* | 275* | 0·01 |
| 20 mm$^3$ | Lung | 275* | 275* | 0·05 |
| 500 mm$^3$ | Flank | 315 (290–347) | 307 (270–357) | 0·36 |

* The data are consistent with the terminal $D_0$ value found for 0·5 mm$^3$ hypoxic tumours.

1–2-mm$^3$ nodules was about two decades greater than in 500-mm$^3$ s.c. tumours. It can be inferred from the shapes of the radiation survival curves for pulmonary tumours (Figs. 3a and 3b) that the change in sensitivity to radiation is largely due to a marked increase in hypoxic fraction, from undetectable levels in 0·5-mm$^3$ nodules to about 0·05 in 15–20-mm$^3$ tumours. These data are summarized in the Table together with our earlier estimate of 0·36 for the hypoxic fraction of 500-mm$^3$ s.c. tumours (Shipley *et al.*, 1975). S.c. and pulmonary tumours of the same size appear to be equally radiosensitive (Fig. 2). It seems unlikely, therefore, that the absence of a detectable hypoxic fraction in 1-mm$^3$ tumour nodules growing in the pulmonary bed is a direct result of the extensive microvasculature of this organ. Furthermore, the proportions of hypoxic cells in lung nodules can be correlated with histological changes: areas of necrosis are not seen in 1-mm$^3$ nodules, but are visible in increasing amounts in 6-mm$^3$ and 20-mm$^3$ tumours.

The response of LL tumours to cytotoxic drugs also shows considerable size-dependence. Figs. 1a and 1b indicate that a marked change in surviving fraction occurs in the size range 1–100 mm$^3$ for both cyclophosphamide and BCNU, whereas the greatest change in sensitivity to radiation appears to take place in pulmonary tumours between 1 and 20 mm$^3$ (Fig. 2). While the emergence of a measurable hypoxic fraction in tumours greater than 5 mm$^3$ explains the radiation results, the reason for size-dependence of response after drug treatment is not clear. Furthermore, unlike radiation treatment, BCNU appears to be more effective against pulmonary tumours than s.c. tumours of the same size (Fig. 1a). A number of studies have evaluated the response of various experimental tumour systems to this drug. Hagemann, Schenken and Lesher (1973) have studied the response of P815 × 2 mastocytoma cells grown in culture, ascites and solid forms. They found that solid tumours were far less sensitive to the drug than either of the other forms and suggested that this was a consequence of the failure of the drug to reach significant numbers of cells at risk. Rosenblum *et al.* (1975) have studied the effect of BCNU on a rat brain tumour and found that the dose-response curve for this drug was biphasic, with little extra cell kill noted at doses greater than 0·75 times the $LD_{10}$. They also considered that accessibility of this drug to clonogenic tumour cells might be the limiting factor in its effectiveness.

Earlier results from this laboratory indicated that BCNU treatment of the murine tumour B16 melanoma left a surviving population that was predominantly hypoxic (Hill and Stanley, 1975*b*), suggesting that radiation and BCNU spare the same sub-population of clonogenic tumour cells. The implication of this is not necessarily that the hypoxic state of the cells is directly responsible for their resistance to BCNU, as the failure of the drug to reach these cells in adequate concentrations would have the same effect.

The radiation dose-response curves from 6-mm$^3$ and 20-mm$^3$ pulmonary tumours have been analysed by comparison with curves predicted by a multi-target single-hit model for a mixed oxic and hypoxic cell population (Elkind and Whitmore, 1967). For both tumour sizes studied, the experimental data fitted quite closely to the terminal region of the theoretical curve predicted by the model (Figs. 3a and 3b). Both sets of data show a gradual inflexion before the radioresistant terminal portion, and for 6-mm$^3$ nodules, surviving fractions between 0·1 and 0·01 lie consistently above the line predicted by the two-component model. These data may be better interpreted in terms of the model proposed by Tannock (1972), which allows for a distribution of radiosensitivities between that of maximally sensitive and that of maximally resistant cells.

A large single dose of cyclophosphamide produced only regression and some growth delay in 16-day pulmonary tumours (Fig. 4), results which are qualitatively similar to those reported by Ovejera, Johnson and Goldin (1975) for the same tumour system. However, we have shown that the pulmonary nodules regrowing after cyclophosphamide treatment appeared to be as radiosentisive as previously untreated tumours (Fig. 5). This indicates that the effect of drug treatment on the tumour cells and stroma did not impair the ability of the surviving population to reoxygenate and proliferate. These observations may be of some clinical relevance in combined modality treatment of pulmonary disease, using chemotherapy to shrink tumour nodules and consequently to reduce the proportion of hypoxic cells present in the tumour. Suit and Maeda (1967), in studies on the mammary carcinoma MDAH-MCa-4 in C3H mice, observed that high pressure $O_2$ administered during radiation treatment was only modestly effective in reducing the tumour-control dose for 250-mm$^3$ tumours, whereas the same treatment was much more effective against 0·6-mm$^3$ nodules. It is possible that a similar difference in response to high pressure $O_2$ may exist between 500-mm$^3$ LL tumours with a 0·36 hypoxic fraction and 6- and 20-mm$^3$ pulmonary tumours with hypoxic fractions of 0·01 and 0·05 respectively.

The principal finding of this study has been that, although pulmonary nodules less than 1 mm$^3$ in volume are markedly more sensitive to cytotoxic therapy than larger tumours, this advantage rapidly decreases over 4–5 volume doublings in the case of radiation treatment, due to developing hypoxia. There appear to be critical size ranges over which cellular sensitivity to drugs and radiation changes. For radiation this takes place between 1 and 10 mm$^3$, the size range over which necrosis first becomes visible, whereas the change in sensitivity to drug treatment occurs in the size range between 1 and 100 mm$^3$. It is not known whether in man the critical size range may be similar; in radiographically demonstrable metastatic disease the size of the smallest pulmonary deposits is considerably larger than the most radiosensitive size reported here. The concept of a critical size range may have important implications both for prophylactic therapy of occult metastatic disease and for combined modality treatment of larger tumour deposits. If chemotherapy can shrink metastatic nodules through this size range, then a useful improvement may be gained in subsequent radiotherapy.

The work contained in this report was partly supported by National Cancer Institute Contract No. NCI-CM-23717.

We acknowledge with gratitude the continuing support and helpful advice of Professor L. F. Lamerton and Professor M. J. Peckham.

### REFERENCES

Bonadonna, G., Brusamolino, E., Valagussa, P., Rossi, A., Brugnatelli, L., Brambilla, C., de Lena, M., Tancini, G., Bajetta, E., Musumeci, R. & Veronesi, U. (1976) Combination Chemotherapy as an Adjuvant Treatment in Operable Breast Cancer. *New Engl. J. Med.*, **294**, 405.

Courtenay, V. D. (1976) A Soft Agar Colony Assay for Lewis Lung Tumor and B16 Melanoma Taken Directly from the Mouse. *Br. J. Cancer*, **34**, 39.

DeWys, W. D. (1972) A Quantitative Model for the Study of the Growth and Treatment of a Tumor and its Metastases with Correlation between Proliferative State and Sensitivity to Cyclophosphamide. *Cancer Res.*, **32**, 367.

Elkind, M. M. & Whitmore, G. F. (1967) *The Radiobiology of Cultured Mammalian Cells.* New York: Gordon and Breach.

Fu, K. K., Phillips, T. L. & Wharam, M. D. (1976) Radiation Response of Artificial Pulmonary Metastases of the EMT6 Tumor. *Int. J. Rad. Oncol. Biol. Phys.*, **1**, 257.

Hagemann, R. F., Schenken, L. L. & Lesher, S. (1973) Tumor Chemotherapy Efficacy Dependent on Mode of Growth. *J. natn. Cancer Inst.*, **50**, 467.

Heyn, R. M., Holland, R., Newton, W. A., Tefft, M., Breslow, N. & Hartmann, J. R. (1974) The Role of Combined Chemotherapy in the Treatment of Rhabdomyosarcoma in Children. *Cancer, N.Y.*, **34**, 2128.

Hill, R. P. & Stanley, J. A. (1975*a*) The Lung Colony Assay: Extension to the Lewis Lung Tumour and the B16 Melanoma-Radiosensitivity of B16 Melanoma Cells. *Int. J. Radiat. Biol.*, **27**, 377.

Hill, R. P. & Stanley, J. A. (1975*b*) The Response of Hypoxic B16 Melanoma Cells to *in vivo* Treatment with Chemotherapeutic Agents. *Cancer Res.*, **35**, 1147.

Hill, R. P. & Stanley, J. A. (1977) Pulmonary Metastases of the Lewis Lung Tumor, their Cell Kinetics and Response to Cyclophosphamide at Different Sizes. *Cancer Treatment. Rept*, **61**, 29.

JAFFE, N., FREI, E., III., TRAGGIS, D. & BISHOP, Y. (1974) Adjuvant Methotrexate and Citrovorum Factor Treatment of Osteogenic Sarcoma. *New Engl. J. Med.*, **291**, 994.

OVEJERA, A. A., JOHNSON, R. K. & GOLDIN, A. (1975) Growth Characteristics and Chemotherapeutic Response of Intravenously Implanted Lewis Lung Carcinoma. *Cancer Chemother. Rep.*, **5**, 111.

ROSENBLUM, M. L. S., WHEELER, K. T., WILSON, C. B., BARKER, M. & KNEBEL, K. D. (1975) *In vitro* Evaluation of *in vivo* Brain Tumor Chemotherapy with 1,3-Bis(2-chloroethyl)-1-nitrosourea. *Cancer Res.*, **35**, 1387.

SHIPLEY, W. U., STANLEY, J. A. & STEEL, G. G. (1975) Tumor Size Dependency in the Radiation Response of the Lewis Lung Carcinoma. *Cancer Res.*, **35**, 2488.

STEEL, G. G. & ADAMS, K. (1975) Stem-cell Survival and Tumor Control in the Lewis Lung Carcinoma. *Cancer Res.*, **35**, 1530.

STEEL, G. G., ADAMS, K. & STANLEY, J. A. (1976) Size Dependence of the Response of Lewis Lung Tumors to BCNU. *Cancer Treatment. Rept.* **60**, 1743.

SUGIURA, K. & STOCK, C. C. (1955) Studies in a Tumor Spectrum, III. The Effect of Phosphoramides on the Growth of a Variety of Mouse and Rat Tumors. *Cancer Res.*, **15**, 38.

SUIT, H. D. & MAEDA, M. (1967) Hyperbaric Oxygen and Radiobiology of a C3H Mouse Mammary Carcinoma. *J. natn. Cancer Inst.*, **39**, 639.

TANNOCK, I. F. (1972) Oxygen Diffusion and the Distribution of Cellular Radiosensitivity in Tumours. *Br. J. Radiol.*, **45**, 515.

TWENTYMAN, P. R. & BLEEHEN, N. M. (1976) The Sensitivity to Cytotoxic Agents of the EMT6 Tumour *in vivo*. Comparative Response of Lung Nodules in Rapid Exponential Growth and of the Solid Flank Tumour. *Br. J. Cancer*, **33**, 320.

VAN DEN BRENK, H. A. S., BURCH, W. H., KELLY, H. & ORTON, C. (1975) Venous Diversion Trapping and Growth of Blood-borne Cancer cells "en route" to the Lungs. *Br. J. Cancer*, **31**, 46.